# Diagnosis and Management of Malignant Pleural Effusion: A Decade in Review

**DOI:** 10.3390/diagnostics12041016

**Published:** 2022-04-18

**Authors:** Blake Jacobs, Ghias Sheikh, Houssein A. Youness, Jean I. Keddissi, Tony Abdo

**Affiliations:** Section of Pulmonary, Critical Care and Sleep Medicine, The University of Oklahoma Health Sciences Center and The Oklahoma City VA Health Care System, Oklahoma City, OK 73104, USA; blake-jacobs@ouhsc.edu (B.J.); ghias-sheikh@ouhsc.edu (G.S.); houssein-youness@ouhsc.edu (H.A.Y.); jean-keddissi@ouhsc.edu (J.I.K.)

**Keywords:** malignant pleural effusion, indwelling pleural catheter, thoracoscopy, chest tube, talc, pleurodesis

## Abstract

Malignant pleural effusion (MPE) is a common complication of thoracic and extrathoracic malignancies and is associated with high mortality. Treatment is mainly palliative, with symptomatic management achieved via effusion drainage and pleurodesis. Pleurodesis may be hastened by administering a sclerosing agent through a thoracostomy tube, thoracoscopy, or an indwelling pleural catheter (IPC). Over the last decade, several randomized controlled studies shaped the current management of MPE in favor of an outpatient-based approach with a notable increase in IPC usage. Patient preferences remain essential in choosing optimal therapy, especially when the lung is expandable. In this article, we reviewed the last 10 to 15 years of MPE literature with a particular focus on the diagnosis and evolving management.

## 1. Background

Malignant pleural effusion (MPE) is a common complication of advanced malignancy. It is associated with increased morbidity, mortality, healthcare utilization, and cost. The incidence of MPE in the US surpasses 150,000 cases per year, with yearly hospitalizations close to 125,000 [1,2]. The median hospitalization’s length of stay is around 6 days, costing around $42,000 per admission [3,4]. The majority of MPE occurs secondary to metastatic disease; lung cancer being the most frequent etiology in males and breast cancer in females [2,5].

The management of MPE remains palliative in nature with a median survival of 3–12 months [5]. Over the last 15 years, clinical research has focused on decreasing hospitalizations and length of stay by shifting the management to the outpatient setting. Effusion drainage and pleurodesis remain the cornerstone of MPE management. The rate of pleurodesis has been the primary outcome for many studies, and improvement in dyspnea is the primary outcome in others. The last decade witnessed a significant increase in the number of randomized controlled trials that better defined MPE management. As a result, updated clinical practice guidelines were published by different respiratory societies in 2018 [6,7]. While individualized targeted therapy that takes into consideration disease heterogeneity is still lacking, a minimally invasive approach focusing on patient preferences, symptoms, quality of life, and lower hospitalizations days should be adopted. This review addresses the diagnosis, prognosis, and management of MPE according to the most current literature.

## 2. Diagnosis

### 2.1. Clinical Presentation

The clinical presentation of MPE is variable. Although dyspnea is the most common symptom, 25% of MPE patients are asymptomatic with incidental findings on exam or radiography [8,9,10]. In addition to compression of lung parenchyma by the effusion, a complex interplay of mediastinal shift, reduced chest wall compliance, diaphragm depression, and reflex stimulation from the lungs and chest wall is likely responsible for the sensation of dyspnea [11,12]. Improvement in these mechanics, rather than just fluid volume removal is responsible for post-thoracentesis relief [12,13]. Other associated symptoms include dull ipsilateral chest pain that signifies malignant involvement of ribs and parietal pleura [14] and constitutional symptoms such as weight loss and cachexia, which are commonly part of the underlying malignancy.

### 2.2. Imaging

#### 2.2.1. Chest Radiography

An abnormal chest radiograph is usually the first investigation in diagnosing MPE. About 15% of MPE patients will have less than 500 mL in volume [1], with the majority being moderate to large effusions. A massive effusion is defined as opacification of an entire hemithorax and is seen in 10% of patients with MPE. However, MPE is the most common cause (67%) of massive effusions [15].

#### 2.2.2. Thoracic Ultrasonography

Ultrasound has a much higher sensitivity for detecting effusions when compared to chest X-ray [16]. There are certain features described on thoracic ultrasound that are suggestive of malignant disease. These include pleural thickening (>1 cm), diaphragmatic thickening (>7 mm), and pleural nodularity [17]. However, as evidenced by a recent systematic review and meta-analysis, thoracic ultrasound is not useful in ruling out MPE [18]. Ultrasonography has become the standard of care for performing diagnostic thoracentesis for MPE, especially with site selection, decreasing complication rate, and evaluating lung re-expansion post drainage [19].

#### 2.2.3. Chest Computed Tomography (CT)

Chest CT is a quick and non-invasive method of providing a detailed image of the pleural space and should be strongly considered in any patient with an undiagnosed unilateral exudative pleural effusion [20]. The main CT findings that suggest malignant pleural mesothelioma include the presence of unilateral effusion, interlobar fissure thickening, and pleural nodularity [21]. A study by Porcel et al. showed that a validated CT scan-based scoring system may predict MPE. The score included pleural lesions >1 cm (5 points), presence of liver metastasis, abdominal mass, or lung nodule >1cm (3 points each); and absence of cardiomegaly, pericardial effusions, or loculations (2 points each). A score ≥7 predicted MPE with excellent sensitivity (88%) and specificity (94%) [22].

#### 2.2.4. PET Scan

PET scan is frequently obtained to stage patients with malignancies, but historically it has had a poor predictive value for MPE due to the increased FDG uptake related to infection and nonspecific inflammation [23]. However, a recently developed and validated PET-CT score may be a useful tool to differentiate malignant from benign effusions. This score awards different points based on FDG avid lung nodules and/or masses (unilateral 3 points, bilateral 1 point), extrapulmonary malignancies (3 points), pleural thickening (2 points), and FDG avid effusion (1 point). A cut-off value of 4 points had a sensitivity of 95% and a specificity of 83% for detecting MPE [24].

### 2.3. Diagnostic Pleural Procedures

#### 2.3.1. Thoracentesis and Pleural Fluid Analysis

Thoracentesis is usually the first performed procedure and should be done under ultrasound guidance. MPEs are overwhelmingly exudative, but transudates can be seen in 5–10% of cases [25]. These transudates are usually related to an underlying condition causing increased hydrostatic pressure, such as congestive heart failure, superior vena cava syndrome, airway obstruction with resultant atelectasis, or low oncotic pressure from underlying malnutrition or cachexia. MPEs often have a low pleural fluid pH (<7.3) and glucose (<60 mg/dL). A low pleural fluid pH has been associated with a higher initial positive yield on cytology [26]. However, a recent large retrospective trial showed that neither pleural fluid pH nor glucose was associated with an increase in the diagnostic yield of pleural fluid cytology. On the other hand, a higher pleural fluid LDH did correlate with a higher diagnostic yield of MPE in this study [27]. Previous reports of a low pH having prognostic value and predictive value for pleurodesis success were also disproved in two studies by Heffner et al. [28,29]. Pleural fluid cell count differential is lymphocyte-predominant in more than 50% of MPEs. The yield of pleural fluid cytology to diagnose MPE on an initial sample is around 60%, increasing by 27% with a second sample [20] with a minimal increase in diagnostic yield on subsequent sampling [30]. Hence, a pleural biopsy would be the next step. However, when pleural fluid cytology is negative but the clinical picture and imaging are highly suggestive of MPE, a pleural biopsy is seldomly pursued as it will unlikely change the management. The diagnostic yield of pleural fluid cytology varies by cancer type with the highest yield in adenocarcinoma (70–95%) and the lowest in mesothelioma (0–6%) [27,31,32]. Within adenocarcinoma, pleural fluid cytology sensitivity is the highest for ovarian cancer (95%) [27]. Cancer biomarkers, while highly specific, have such poor sensitivity that no single marker is considered accurate enough for routine use. The volume of fluid analyzed also improves diagnostic yield, but only up to 75 mL when using direct smear alone [33]. Adding cell block to the smear has a complementary effect on diagnostic sensitivity [34], but the recommended volume to do both is 150 mL [35].

#### 2.3.2. Pleural Biopsy: Blind and Image-Guided

Blind pleural biopsy is performed using an Abrams needle and has significant drawbacks mostly related to low sensitivity in MPE and complications rate. Blind pleural biopsy has a lower diagnostic sensitivity for MPE compared to pleural fluid cytology. It only increases the diagnostic sensitivity by 7–15%, when combined with pleural fluid cytology. Complications such as pneumothorax are common with blind biopsies (3–9%) [36,37].

Contrast-enhanced chest CT and ultrasound-guided pleural biopsies have a much higher sensitivity than blind biopsy for diagnosing malignancy, especially in the presence of pleural thickening. In a randomized trial, CT-guided biopsy had a sensitivity of 87% compared to 40% for Abram’s biopsy [38]. An ultrasound-guided pleural biopsy had a sensitivity of 70% for malignancy compared to 40% for blind biopsy [39]. In another study by Hallifax et al., the diagnostic yield of ultrasound-guided pleural biopsy for malignancy was around 94% [40]. Given these findings, blind biopsies for MPE diagnosis should be discouraged.

#### 2.3.3. Medical Thoracoscopy and Video-Assisted Thoracoscopic Surgery

Pleural biopsies done under direct visualization via medical thoracoscopy (MT) or video-assisted thoracoscopic surgery (VATS) allow for even higher diagnostic sensitivity given the ability to localize areas with pleural nodularity. An analysis pooling 22 case series involving MT with local anesthesia showed 92.6% diagnostic sensitivity for pleural malignancy. MT sensitivity remained around 90% even when narrowed to patients where initial closed bind biopsy was negative [41]. There has not been a head-to-head comparison of MT against VATS for MPE diagnosis. Both offer diagnostic as well as therapeutic avenues with VATS offering the advantage of more effective drainage of tenuous loculations with the option to convert to open thoracotomy if necessary [42].

Pleural biopsy is a preferred diagnostic method over fluid cytology when mesothelioma is suspected [5,43]. Early local radiotherapy was recommended to prevent malignant seeding after invasive diagnostic procedures in patients with malignant pleural mesothelioma [44], but two large multicenter randomized controlled trials showed that prophylactic radiotherapy is not needed [43,45,46].

## 3. Prognosis

Patients presenting with MPE have an overall poor prognosis with a median survival of 3 to 12 months [5,9]. Predicting MPE prognosis may allow for a better selection of the optimal treatment.

Primary tumor type affects prognosis. MPEs associated with chemoresponsive tumors, such as hematologic tumors and breast cancers, have prolonged survival, while patients with MPEs due to lung, gastrointestinal, urologic, and sarcomatous malignancies have a lower survival [47]. Mesothelioma is a common cause of MPE conferring a relatively long median survival of 8–12 months but with wide variability among patients. Brims et al. developed and validated a clinical prediction model for malignant pleural mesothelioma that defined four risk groups with clear survival differences (median survival of 34, 17.7, 12, and 7.4 months, respectively). Weight loss was the strongest predictive variable. Other variables included hemoglobin, albumin, eastern cooperative oncology group (ECOG) performance status, and histological diagnosis [48].

Clinical factors including performance status scores (Karnofsky or Eastern Cooperative Oncology Group (ECOG)), breathlessness, hypoxemia, and weight loss have been shown to significantly affect prognosis [49,50,51,52]. Systemic markers of inflammation such as elevated C-reactive protein, neutrophil-to-lymphocyte ratio, platelet-to-lymphocyte ratio, leukocytosis, and decreased serum albumin have consistently been shown to signal worse prognosis [51,52,53]. However, there is conflicting evidence regarding the utility of pleural fluid analysis to determine prognosis, with elevated pleural fluid LDH and low pleural fluid protein often conferring a worse prognosis, while pleural fluid pH and glucose levels have variable reported utility [49,52,54].

The LENT score published in 2014 was the first validated score for predicting survival in malignant pleural effusion. The LENT score incorporates four variables (pleural fluid LDH level, ECOG performance status, blood neutrophil-to-lymphocyte ratio, and tumor type) and divides patients into low (score 0–1), moderate (score 2–4), or high (5–7) risk with a median survival of 319, 130, and 44 days, respectively [47].

A prospectively validated score (PROMISE) published in 2018 predicted survival better than the LENT score. This was the first prognostic score to combine biological markers and clinical parameters. The PROMISE score includes seven variables (chemotherapy, radiotherapy, hemoglobin, white blood cell count, C-reactive protein, ECOG performance status, and cancer type) in addition to pleural fluid tissue inhibitor of metalloproteinases 1 (TIMP1) for the biological PROMISE score. This score stratifies patients based on 3-month mortality risk into one of four groups (A < 25%, B 25% to <50%, C 50% to <75%, and D ≥ 75%) (Table 1) [55]. Despite performing better than the LENT score, the complexity of the PROMISE score continues to be a limiting factor to its daily practice. In fact, the role of both scores in clinical practice is still unclear, and future studies are needed to determine if their use correlates with any improvement in patient-centered outcomes.

## 4. Management of MPE

### 4.1. Asymptomatic Malignant Pleural Effusion

The first step in the management of malignant pleural effusion is to determine the effusion’s impact on the patient’s quality of life. Asymptomatic MPE, particularly in a patient with a limited life expectancy, can be reasonably observed without any intervention [7]. MPEs secondary to chemoresponsive malignancies respond to systemic therapy without requiring direct intervention. Incidentally noted MPEs considered too small to safely undergo thoracentesis have been shown to remain stable and not require intervention [56,57]. However, these effusions were associated with a worse prognosis compared to patients without MPE [57]. In a recent multicenter retrospective study that excluded minimal effusion (<10 mm depth) and patients who died within 3 months of diagnosis, 41% of patients with asymptomatic MPE secondary to nonsmall lung cancer became symptomatic within 1 year, with a median time to symptoms development of 4 months. Female sex and MPE size were independently associated with future development of symptoms requiring intervention [10].

### 4.2. Therapeutic Thoracentesis

Large-volume thoracentesis is widely considered the first step in the management of malignant pleural effusion [7]. It allows for evaluation of symptom improvement, rate of reaccumulation, and the re-expandability of the lung, all of which inform further management options [12,13,58]. The routine use of ultrasound guidance for thoracentesis is recommended as pooled data from multiple studies have shown that the safety of thoracentesis is improved with a lower associated cost [59,60].

Symptom improvement from MPE drainage is often dramatic and out of proportion to the improvement in spirometry and oxygenation. A recent study (PLEASE) that prospectively enrolled patients with symptomatic pleural effusion showed a significant improvement in breathlessness and exercise tolerance after therapeutic thoracentesis. Breathlessness improvement was similar in participants with and without trapped lung [13]. While the dyspnea associated with MPE appears to be multifactorial, an important component appears to be pressure on the diaphragm and chest wall causing abnormal hemidiaphragm shape and movement. This has been theorized to cause neuromechanical uncoupling whereby the ventilatory output is decreased relative to neural drive causing the sensation of breathlessness [12,13].

The MPE reaccumulation rate is another important consideration. Risk factors associated with MPE recurrence are unclear. A recent retrospective multicenter cohort study evaluated around 1000 patients following MPE drainage and found that 30% of patients experienced recurrence by day 15 and 48% by day 90. Larger effusion and higher pleural fluid LDH correlated with increased recurrence, while negative cytology was associated with decreased hazard of recurrence [58]. In patients with a very poor prognosis and predicted survival in weeks, a one-time thoracentesis may be all that is needed to remove the effusion.

The first large volume thoracentesis is also useful for determining if the lung is re-expandable. Pleural manometry can be used to predict the presence of a non-expandable lung; however, a recent prospective multicenter randomized controlled trial by Lentz et al. did not support the routine use of pleural manometry during large volume therapeutic thoracentesis (no difference in procedure-related chest discomfort) [61]. However, pleural manometry may still have some value in predicting non-expandable lung, pleurodesis success, and potentially assigning patients with MPE to the right management pathway as suggested by the EDIT (Elastance-Directed Intrapleural Catheter or TALC pleurodesis in Malignant Pleural Effusion) management feasibility trial; patients with high pleural elastance were allocated to IPC and others to talc pleurodesis [62]. However, a recent small single-center retrospective trial showed a significant discordance between pleural elastance and post-thoracentesis radiograph (complete lung expansion on chest radiograph with elevated pleural elastance or incomplete lung expansion on chest radiograph with normal elastance) [63].

### 4.3. Pleurodesis

Pleurodesis is the obliteration of the pleural space by inducing inflammation, scarring, and fusion of the visceral and parietal pleura. Mechanical and/or chemical pleurodesis is often a definitive treatment for malignant pleural effusions. Chemical pleurodesis, which includes administration of sclerosant agents mostly through a chest tube, requires pleural apposition and is not an option for patients with non-expandable lung. Until recently, pleurodesis was performed almost exclusively in the inpatient setting, requiring a significant amount of time and resources with the hope of decreasing future interventions and hospitalizations.

Graded sterile talc instilled via thoracoscopy (talc poudrage) or through a chest tube in suspension form (talc slurry) is the preferred agent for chemical pleurodesis with a lower pleurodesis failure rate compared to many other agents including doxycycline and bleomycin [64]. The early use of ungraded talc as a sclerosant agent was associated with cases of pleurodesis-induced acute respiratory distress syndrome [65,66], prompting a shift towards graded talc with significant improvement in safety [67].

Several factors have been shown to affect the success of talc pleurodesis. Systemic corticosteroids have been shown to decrease pleurodesis success [68]. Systemic nonsteroidal anti-inflammatory drugs (NSAIDs) were linked to a lower pleurodesis success in earlier studies, but a large randomized controlled trial (TIME1) showed no difference in 3-month pleurodesis rate when compared to opioids. TIME1 also compared small-bore (12 French) with large-bore (24 French) chest tubes showing a higher pleurodesis failure rate with small-bore chest tubes (30%) compared to large bore (24%), but small-bore chest tubes were less painful [69]. It is unclear if the lower pleurodesis rate associated with small-bore is related to the size of the tube itself or more so to the higher rate of occlusion. A recent metanalysis showed no difference in pleurodesis rate between small- and large-bore chest tubes [70].

The question of the effectiveness of talc poudrage administered via thoracoscopy versus talc slurry administered via chest tube has been investigated in multiple studies, often with a statistically insignificant trend toward improved pleurodesis rate with talc poudrage but with a trend towards more adverse events [6,64,65,71]. In a large randomized controlled trial, Dressler et al. compared thoracoscopy-guided talc poudrage to talc slurry and showed no overall difference in 30-day pleurodesis rates. In a subgroup analysis, talc poudrage was associated with a higher pleurodesis rate compared to talc slurry (82% vs. 67%) among patients with either lung or breast cancer. The American Thoracic Society, Society of Thoracic Surgeons, and Society of Thoracic Radiology combined guidelines (ATS/STS/STR) published in 2018 suggest the use of either talc poudrage or talc slurry in patients with MPE undergoing pleurodesis (conditional recommendation, low confidence in estimate of effects) [7]. A recently published multicenter randomized controlled trial (TAPPS) tested the hypothesis that administration of talc poudrage during thoracoscopy under moderate sedation is more effective than talc slurry via chest tube in successfully inducing pleurodesis. The study showed no difference in the 90-day pleurodesis failure rate (22% in talc poudrage vs. 24% in talc slurry) and no statistically significant differences in any of the 24 prespecified secondary outcomes, including pleurodesis rate at 30 and 180 days, length of hospital stay, mortality, chest pain, dyspnea, and quality of life. Notably, two thirds of patients enrolled had either breast or lung cancer [71].

A recent randomized controlled trial (SIMPLE) investigated whether the use of thoracic ultrasonography in pleurodesis pathways could shorten hospital stay in patients with MPE compared to usual care per the British Thoracic Society guidelines (based on daily output volume). Thoracic ultrasonography-guided care resulted in shorter hospital stays (median difference of 1 day) with no reduction in the procedure’s success rate at three months [72].

Another common question is whether the use of intrapleural fibrinolytics can be used to improve drainage of loculated MPEs to achieve pleural apposition and subsequently improve pleurodesis rate. Early small randomized controlled trials pointed out that improvement in imaging, fluid output, and dyspnea constitute potential evidence that intrapleural fibrinolytics were helpful in loculated MPEs despite some not showing a statistically significant improvement in pleurodesis success rate [73,74]. Further investigation has shown that fibrinolytics likely induce pleural fluid production by mesothelial cells, possibly via monocyte chemotactic protein (MCP-1) production, calling into question the use of fluid output as a meaningful marker of improvement [75]. The TIME3 trial, a prospective double-blind placebo-controlled randomized trial, assessed the effect of intrapleural urokinase on dyspnea and pleurodesis in patients with septated MPE. The study showed no difference in mean dyspnea or pleurodesis failure rate over 12 months (urokinase 37% vs. placebo 32%). Intrapleural urokinase was associated with significant improvement in chest radiograph appearance, reduced length of hospital stays, and decreased mortality [76]. These secondary outcomes need further exploration. Urokinase has no antitumor effect, and overall survival was very low in this study (median survival for the urokinase group was 69 days vs. 48 days for placebo). Intrapleural fibrinolytics should not be routinely used in such patients but might have a role in those with prolonged survival. Thoracoscopy or indwelling pleural catheter (with possibly intrapleural fibrinolytics) may be better alternatives in septated MPE.

### 4.4. Indwelling Pleural Catheter (IPC)

An IPC is a 15.5 French silicone tube, inserted into the pleural space, and tunneled under the skin for long-term outpatient management of MPE. Since its approval by the FDA in 1997, IPC use has been on the rise with a dramatic increase between 2008 and 2012 after which it leveled off [77,78]. Putnam et al. published the first randomized controlled trial comparing IPC to doxycycline pleurodesis in 1999 and showed a significant decrease in hospitalization days in the IPC group with a similar degree of symptomatic improvement and quality of life [79]. Between 2000 and 2010, multiple case series were published showing improvement in symptoms with IPC without major complications and a spontaneous pleurodesis rate of around 45% [77]. The TIME 2 trial, a multicenter randomized controlled trial in the United Kingdom published in 2012, compared IPC to chest tube and talc pleurodesis for relieving dyspnea in patients with MPE and showed no difference in dyspnea at 6 weeks. Notably, the IPC group was associated with a lower dyspnea score at 6 months and a shorter hospital stay. No difference in chest pain, quality of life, serious adverse events, or mortality was seen [80]. The AMPLE (Australian Malignant Pleural Effusion) multicenter randomized controlled trial, published in 2017, also looked into IPC vs. talc pleurodesis in patients with MPE but with hospitalization days as the primary outcome. Patients in the IPC group spent a median of 2 days less in the hospital from procedure to death or to 12 months (10 days in the IPC group vs. 12 days in talc pleurodesis). This reduction in hospitalization days was mostly related to the reduction in effusion-related hospitalization days (median of 1 day for IPC group vs. 4 days with pleurodesis). Patients in the pleurodesis group required a second intervention (usually an IPC insertion) 18% more compared to patients in the IPC group. No difference was seen in breathlessness or quality of life [81]. The 2010 British Thoracic Society guidelines for MPE management recommended IPC as a second-line treatment for refractory MPE failing conventional pleurodesis or for non-expandable MPE [9]. With growing evidence over the last decade supporting IPC use for MPE management, the recent ATS/STS/STR guidelines recommended using either an IPC or chemical pleurodesis in symptomatic patients with MPE and suspected expandable lung and IPC for patients with MPE and non-expandable lung [7,80,81].

In 2017, two multicenter randomized controlled trials looking for the optimal regimen of drainage after IPC insertion were published. The ASAP trial compared an aggressive (daily) drainage to a standard (every other day) regimen with the incidence of auto-pleurodesis (complete or partial) as the primary outcome. At 12 weeks, the rate of auto-pleurodesis was greater in the aggressive drainage arm than in the standard drainage arm (47% vs. 24%, respectively) with a shorter median time to autopleurodesis in the aggressive arm (54 days) compared to the standard arm (90 days) [82]. In the AMPLE-2 trial, patients were randomized to either an aggressive (daily) drainage regimen or symptom-based. The primary outcome was the mean daily breathlessness score measured during the first 60 days, and secondary outcomes included spontaneous pleurodesis rate and quality of life. There was no difference in mean daily breathlessness score between the two groups, but a trend toward improved quality of life in the aggressive drainage group was seen. The rate of spontaneous pleurodesis was higher with aggressive/daily drainage (37% at 60 days, 44% at 6 months) compared to symptom-guided (11% at 60 days, 16% at 6 months). The lower pleurodesis rate in this trial compared to previous ones may be explained by the fact that one third of the enrolled patients had non-expandable lung [83]. Based on these two studies, daily drainage may benefit patients with expandable lungs, although this strategy may be more labor-intensive, require more supplies, and be associated with increased healthcare costs.

Talc pleurodesis through the IPC is an exciting clinical pathway that allows complete ambulatory management of the MPE while still considering pleurodesis as an option. A small retrospective case series by Ahmed et al. suggested the efficacy and safety of administering talc slurry through the IPC in the outpatient setting with a very high rate of pleurodesis success (92% at 14 days) in a highly selected group of patients with complete lung expansion confirmed by ultrasonography or chest tomography [84]. The IPC-PLUS trial was the first randomized controlled trial to explore the outpatient administration of talc through IPC with the hypothesis that it will increase pleurodesis rate compared to IPC alone. Patients were recruited from 18 centers across the United Kingdom. The IPC was inserted under local anesthesia, maximal fluid drained, and the patient was discharged home with instructions to follow up on day 10 when randomization to placebo or talc occurred if more than 75% pleural apposition was seen on the chest radiograph. The talc administration through the IPC resulted in a significantly higher chance of pleurodesis at 35 days compared to placebo (43% vs. 23%, respectively), with no increase in adverse events. At day 70, the pleurodesis rate increased to 51% in the talc group compared to 27% in the placebo [85]. However, the results of this trial may only apply to a selected group of patients with MPE given the high number of patients excluded before randomization. The rate of successful pleurodesis was also lower compared to chest drain and talc pleurodesis. This may be explained by the inability to maintain a dry pleural space in the outpatient setting, especially with the thrice-weekly drainage regimen and the inclusion of patients with partial lung expansion. The EPIToME (Early Pleurodesis via IPC with Talc for Malignant Effusion) observational trial showed that a strategy of using the IPC as the first-line definitive therapy for all patients, followed by inpatient talc pleurodesis through the IPC in patients with expandable lungs, and daily drainage is reasonable. Pleurodesis was achieved in 74% of patients at a median of 20 days. However, around half of patients were not candidates for pleurodesis through the IPC [86]. A study (ASAP-II) randomizing patients with MPE to IPC with talc and daily drainage vs. daily drainage alone is currently recruiting (expected completion 2024) [87]. Confirmation of complete lung expansion pre-enrollment and aggressive daily drainage may lead to a higher pleurodesis rate in the outpatient setting.

Drug-eluting IPCs were promising after a small pilot study showed no adverse effects with silver nitrate-coated IPCs [88]. However, a phase 3 trial (SWIFT) investigating silver nitrate-coated IPCs reported lower pleurodesis rate and more adverse events with the silver nitrate-coated IPCs [89].

Rapid pleurodesis combines thoracoscopic-guided talc poudrage with IPC insertion during the same procedure. This method was associated with a median hospital stay of 2 days and a median duration of IPC of 10 days [90]. In a recently published single-center retrospective chart-based study, ambulatory thoracoscopic poudrage and IPC insertion were found to be a safe and effective option in the management of MPE, with 86.7% of patients discharged the day of the procedure and a 77.8% pleurodesis rate at 6 months [91]. A multicenter randomized controlled trial (TACTIC) comparing thoracoscopy, talc poudrage, and IPC with outpatient management vs. thoracoscopy and poudrage with admission for a chest drain, should start recruiting soon [92].

Penz et al. compared costs associated with IPC vs talc pleurodesis using data from the TIME2 clinical trial and showed no significant difference in the cost. Nevertheless, for patients with expected limited survival of less than 14 weeks, IPC appears to cost less when nursing care is not needed for drainage [93]. This cost analysis was developed based on the United Kingdom healthcare system and may not apply to other healthcare systems. In a recently published cost-effectiveness analysis using theoretical event probability data derived from the ASAP, AMPLE-2, and IPC-Plus trials in addition to Medicare reimbursement data for cost estimation, Shafiq et al. found that in patients with MPE and expandable lung, IPC + talc may be cost-effective relative to symptom-guided drainage, and daily IPC drainage was not a cost-effective strategy in any scenario (expandable and non-expandable lung) [94]. In another cost-utility analysis including thoracoscopic poudrage with IPC, rapid pleurodesis protocol remained cost-ineffective compared to IPC or talc slurry pleurodesis [95].

Unlike traditional pleurodesis strategies, IPC placement can be done on an outpatient basis but requires more patient education and routine maintenance by the patient or caretaker. IPC has been associated with more adverse events such as pleural infection, cellulitis, and catheter blockage. Hence the need to consider patient preferences, support systems, and relative risks and benefits of IPC vs. pleurodesis, especially when managing patients with MPE and expandable lung, given that both treatment options are valid in this population [7].

### 4.5. IPC-Related Complications

IPC-related complications include infection, catheter tract metastasis, catheter blockage or loculations, catheter dislodgement, and catheter fracture. Cumulative data over the last 10 years have led to a better understanding and management of these complications.

*Infection:* The overall rate of IPC-related infection is around 5%, but infection-related mortality is only 0.3% [7,96,97,98]. These infections have been classified into local IPC-related infections, including cellulitis around the catheter site or tunnel tract, and pleural space infection [96,99]. Predominantly, IPCs do not need to be removed when infection occurs. Management will include antibiotherapy for superficial and deep-seated pleural space infections, and continuous drainage for deep-seated pleural infections, with instillation of fibrinolytics and DNase via the catheter in selective cases, such as in inadequate drainage, before considering IPC removal. Differentiation of a colonized IPC from a true pleural space infection is essential, and clinical correlation with biochemical markers and pleural fluid studies is key. Some clinicians are in favor of sampling pleural fluid through a thoracentesis instead of the IPC, but the optimal method is unclear, and further studies are needed [96]. Minimizing the risk of infection is important. A quality improvement intervention that limited IPCs insertion to the endoscopy suite, with full draping and strict adherence to sterile protocol and preoperative antibiotic administration, decreased the risk of IPC-related infections from 8.2% preintervention to 2.2% postintervention. However, 60% of physicians do not use a preoperative antibiotic, and a recent Delphi consensus statement did not support routine administration of preoperative antibiotics [99]. Education of patients and family members on how to appropriately access the IPC is likely an effective preventive strategy. In patients receiving chemotherapy, IPCs should not be removed for the purpose of reducing the risk of infection given that reported infection rates are similar to those with IPCs not undergoing chemotherapy [96,97,100].

*Catheter-related tract metastasis:* This is a rare complication (<1%) in nonmesothelioma-related IPC insertion, although it is potentially underreported [97,101,102]. Treatment is entirely palliative with analgesia and radiotherapy if deemed needed, with no need to remove the IPC.

*Catheter blockage and loculation:* IPC-related loculations occur in 10% of patients and occur about 2 months after IPC insertion [103]. In a multicenter retrospective review, intrapleural fibrinolytic agents increased fluid drained, with improvement in dyspnea and chest radiograph. Unfortunately, symptomatic loculations recurred in almost half of the patients [104]. Catheter blockage incidence is around 5% and is treated by flushing the catheter with saline and if saline is unsuccessful then with fibrinolytic [96,99,103]. Removal of nondraining IPC with replacement of a new IPC or pleural aspiration of a different pocket is the next step if intrapleural fibrinolytic fails to restore flow. Intrapleural fibrinolytic was complicated by bleeding in 3-5% of the cases, and concurrent anticoagulation may not increase the bleeding risk [104,105].

*Catheter fracture:* Catheter fracture mostly occurs at the time of IPC removal. Incidence is around 10%, with recent data suggesting that this complication is more frequent with Rocket Medical IPC compared to PleurX IPC [106,107,108]. When this occurs, the retained portion of the IPC should be left in place, with no aggressive attempts to remove it [97,106,108].

## 5. Future Directions

**Figure 1:**
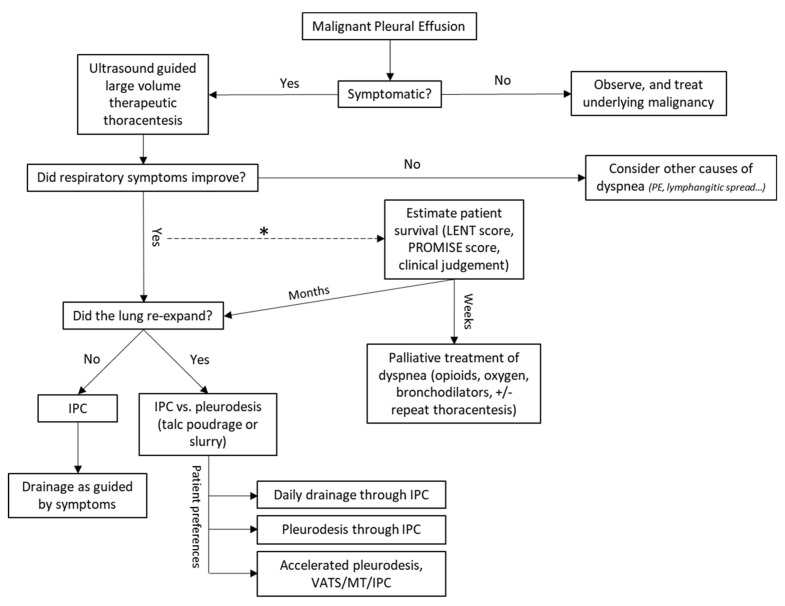
Suggested MPE Management algorithm (adapted from Feller-Kopman et al. [7]). IPC (indwelling pleural catheter), VATS (video assisted thoracoscopic surgery), MT (medical thoracoscopy). * The role of prognostication scores in clinical practice is still unclear, and physicians’ prediction of survival is not very accurate; therefore, a “predicted” short survival should be interpreted with caution.

**Figure 2:**
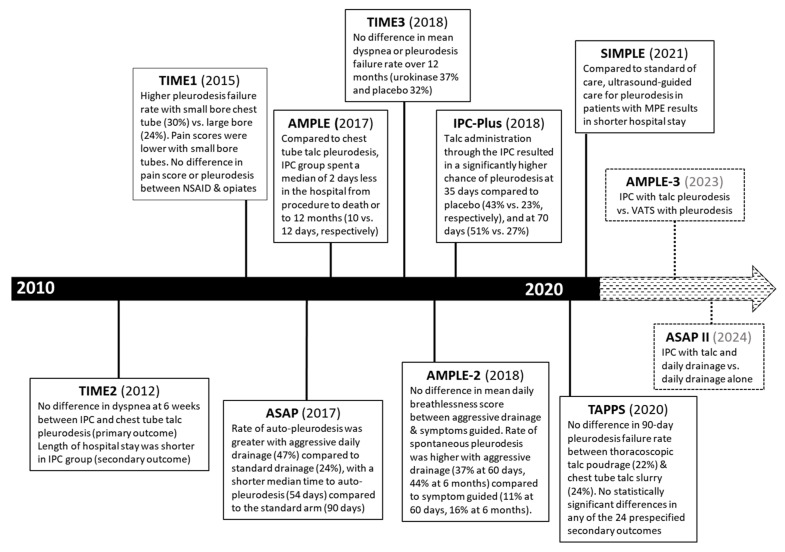
Key randomized controlled trials in MPE management over the last decade. NSAID (nonsteroidal anti-inflammatory drug), IPC (indwelling pleural catheter), MPE (malignant pleural effusion).

Over the last decade, randomized controlled trials and clinical practice guidelines better defined MPE management. Nevertheless, patient preference remains a key when choosing between IPC or chemical pleurodesis in patients with suspected expandable lung (Figure 1). Accelerated autopleurodesis through the IPC has been gaining attention, with the IPC-PLUS trial being the first randomized controlled trial to investigate talc instillation through IPC with good outcomes. The EPIToME observational trial looked into early pleurodesis through the IPC with encouraging results. Ongoing (AMPLE-3 and ASAP II) and soon-to-start (TACTIC) randomized controlled trials are also examining instillation of talc via the IPC and comparing different methods of rapid pleurodesis (Figure 2) [87,92,109]. In addition, a single-center study investigating doxycycline instillation through IPC is currently recruiting [110].

In the era of genotyping and phenotyping every disease and targeted immunological and molecular therapy, malignant pleural effusion should be no exception. MPE is a heterogeneous disease given its association with different types of malignancies, and future research should address it accordingly. This heterogeneity exists even within the same type of cancer. For example, in a study including lung adenocarcinoma with pleural metastasis, epidermal growth factor receptor (EGFR) mutation status was discordant between primary tumors and corresponding pleural metastases in 16% of patients [111]. Translational research over the last decade led to a better understanding of the pathogenesis of MPE, but the clinical application still lags behind, and most available data are limited to phase I trials [112,113]. Intrapleural chemotherapies and immunotherapies have been investigated with conflicting results, and none is ready for prime time yet [113,114,115]. The next decade will surely bring more insights into the pathogenesis of MPE and the role of intrapleural chemotherapy or targeted immunotherapy. Still, it remains unclear to what extent this will affect MPE management.

## Figures and Tables

**Table 1:** Clinical and biological PROMISE scores (adapted from Psallidas et al. [55]).

Variable		Points	
		Clinical PROMISE Score	Biological PROMISE Score
Previous chemotherapy	No	0	0
	Yes	4	3
Previous radiotherapy	No	0	0
	Yes	2	2
Hemoglobin (g/dL)	≥16	0	0
	14 to <16	1	1
	12 to <14	2	2
	10 to <12	3	3
	<10	4	4
Serum white blood cell count (10⁹ cells/L)	<4	0	0
	4 to <6.3	2	2
	6.3 to <10	4	4
	10 to <15.8	7	7
	≥15.8	10	9
C-reactive protein (IU/L)	<3	0	0
	3 to <10	3	3
	10 to <32	5	5
	32 to <100	8	8
	≥100	11	10
ECOG performance status	0–1	0	0
	2–4	7	7
Cancer type	Mesothelioma	0	0
	All other types of cancer	4	5
	Lung	5	6
TIMP1 (ng/mg protein)	<40	n/a	0
	40 to <160	n/a	1
	≥160	n/a	2
**Total score and corresponding 3-month mortality**			
Group (3-month mortality)		Clinical score	Biological score
A: <25%		0–20	0–20
B: 25% to <50%		21–27	21–28
C: 50% to <75%		28–35	29–37
D: ≥75%		>35	>37

## Data Availability

No new data were created or analyzed in this study. Data sharing is not applicable to this article.
